# Impulsivity in abstinent alcohol and polydrug dependence: a multidimensional approach

**DOI:** 10.1007/s00213-016-4245-6

**Published:** 2016-02-25

**Authors:** Eleanor M. Taylor, Anna Murphy, Venkat Boyapati, Karen D. Ersche, Remy Flechais, Shankar Kuchibatla, John McGonigle, Anotonio Metastasio, Liam Nestor, Csaba Orban, Fillippo Passetti, Louise Paterson, Dana Smith, John Suckling, Roger Tait, Anne R. Lingford-Hughes, Trevor W. Robbins, David J. Nutt, JF William Deakin, Rebecca Elliott, ICCAM Platform

**Affiliations:** Neuroscience and Psychiatry Unit, Institute of Brain Behaviour and Mental Health, University of Manchester, Manchester, UK; Behavioural and Clinical Neuroscience Institute, University of Cambridge, Cambridge, UK; Department of Psychiatry, University of Cambridge, Cambridge, UK; Centre for Neuropsychopharmacology, Division of Brain Sciences, Imperial College London, London, UK; GlaxoSmithKline Clinical Research Unit, Cambridge, UK; Department of Psychology, University of Cambridge, Cambridge, UK

**Keywords:** Addiction, Alcohol, Cognition, Drug Abuse, FMRI

## Abstract

**Rationale:**

Dependence on drugs and alcohol is associated with impaired impulse control, but deficits are rarely compared across individuals dependent on different substances using several measures within a single study.

**Objectives:**

We investigated impulsivity in abstinent substance-dependent individuals (AbD) using three complementary techniques: self-report, neuropsychological and neuroimaging. We hypothesised that AbDs would show increased impulsivity across modalities, and that this would depend on length of abstinence.

**Methods:**

Data were collected from the ICCAM study: 57 control and 86 AbDs, comprising a group with a history of dependence on alcohol only (*n* = 27) and a group with history of dependence on multiple substances (“polydrug”, *n* = 59). All participants completed self-report measures of impulsivity: Barratt Impulsiveness Scale, UPPS Impulsive Behaviour Scale, Behaviour Inhibition/Activation System and Obsessive-Compulsive Inventory. They also performed three behavioural tasks: Stop Signal, Intra-Extra Dimensional Set-Shift and Kirby Delay Discounting; and completed a Go/NoGo task during fMRI.

**Results:**

AbDs scored significantly higher than controls on self-report measures, but alcohol and polydrug dependent groups did not differ significantly from each other. Polydrug participants had significantly higher discounting scores than both controls and alcohol participants. There were no group differences on the other behavioural measures or on the fMRI measure.

**Conclusions:**

The results suggest that the current set of self-report measures of impulsivity is more sensitive in abstinent individuals than the behavioural or fMRI measures of neuronal activity. This highlights the importance of developing behavioural measures to assess different, more relevant, aspects of impulsivity alongside corresponding cognitive challenges for fMRI.

**Electronic supplementary material:**

The online version of this article (doi:10.1007/s00213-016-4245-6) contains supplementary material, which is available to authorized users.

## Introduction

Impulsivity is action without forethought, involves premature responding, poor response inhibition and low tolerance for delay (Evenden 1999). It is frequently associated with substance dependence (Dalley et al. 2011; de Wit 2009; Perry and Carroll 2008; Verdejo-García et al. 2008) and higher impulsivity is related to polydrug use (McCown 1988; Semple et al. 2005). Although humans use many different substances legally and illegally, heroin, cocaine and alcohol are rated as the most harmful in the UK (Nutt et al. 2010); these were the focus of the present study.

When investigating the link with substance dependence, impulsivity has been measured in many ways, using either self-report questionnaires or behavioural measures (Verdejo-García et al. 2008). However, self-report and behavioural measures are rarely correlated (Bari and Robbins 2013; Broos et al. 2012), as each measure looks at distinct attributes, often conceptualised in very different ways. Self-report measures, such as the widely used Barratt Impulsiveness Scale (BIS-11; Patton et al. 1995), are assumed to be relatively stable trait constructs, whilst behavioural measures are dependent on specific strategies that may differ between individuals and testing sessions (Bari and Robbins 2013). Whilst self-report measures may be more ecologically valid, they are reliant on individual insight and are susceptible to bias (Verdejo-García et al. 2008).

Two commonly used cognitive paradigms are the Go/NoGo Task (GNG), which measures the ability to inhibit a response before it is initiated, and the Stop Signal Task (SST), which measures inhibition of a response after it is initiated. Both tasks have revealed decreased inhibitory control in cocaine dependence (Ersche et al. 2011; Fernández-Serrano et al. 2012; Kaufman et al. 2003) and alcohol dependence (Sjoerds et al. 2014). A recent meta-analysis of these tasks (Smith et al. 2014) found decreased inhibitory control in alcohol and cocaine dependence, but not in opioid dependence. However, there were very few studies using the GNG task in opioid users and none using the SST. One study since has used the SST in opioid dependence, finding increased impulsivity (Liao et al. 2014).

Studies of the neural substrates of impulsivity emphasise the importance of top-down control of subcortical structures, such as the nucleus accumbens (ventral striatum) by frontal brain regions particularly the orbitofrontal cortex (OFC), anterior cingulate cortex (ACC) and dorsolateral prefrontal cortex (DLPFC; Aron et al. 2003; Hester and Garavan 2004; Kaufman et al. 2003). The inferior frontal gyrus (IFG), especially right sided, ACC and DLPFC are frequently implicated in SST and GNG tasks (Chambers et al. 2009; Garavan et al. 2006; Simmonds et al. 2008). Reduced activations associated with poorer inhibitory control in stimulant users have been observed in the ACC and pre-supplementary motor area (preSMA; Kaufman et al. 2003; Hester and Garavan 2004; Li et al. 2008), as well as the right superior frontal gyrus (Hester and Garavan 2004) and right insula (Kaufman et al. 2003). Reduced prefrontal activation associated with decreased inhibitory control has also been observed in alcohol dependent individuals (Li et al. 2009), whilst neuroimaging studies comparing opioid dependent individuals to controls have found performance impairments accompanied by reduced prefrontal, insula and limbic system responses (Forman et al. 2004; Fu et al. 2008).

Recent investigations of impulsivity pay particular attention to the multifaceted nature of the construct and suggest that different forms of impulsivity are influential at different stages of dependence. For example, impulsive choice (measured using delay discounting and Iowa Gambling Task measures) predicts relapse, whilst impulsive action (measured using SST) does not differentiate abstinent and relapsed participants (Stevens et al. 2015). High impulsive choice is associated with continued drug use and poor maintenance of abstinence (Passetti et al. 2008; MacKillop and Kahler 2009; Washio et al. 2011; Stevens et al. 2013; Stevens et al. 2014), whilst impulsive action is thought to be related to initial sensitivity (Diergaarde et al. 2008; Broos et al. 2012). Stevens et al. (2015) also suggest that behavioural measures are more useful than trait measures for detecting relapse risk, and imply that the different types of measures may be more useful at the different stages of addiction.

A key question is the extent to which impulsivity varies with abstinence. There is evidence for recovery of executive functioning during abstinence (Sullivan et al. 2000; Schulte et al. 2014; Stavro et al. 2013; Fernández-Serrano et al. 2011), as well as more specific evidence for normalisation of behavioural inhibitory control (Hopwood et al. 2011; Morie et al. 2014; Bell et al. 2014). However, findings are not consistent, with other studies reporting that behavioural impulsivity is still elevated in abstinent alcohol dependent participants with mean length of abstinence of six months (Naim-Feil et al. 2014). It has been suggested that abstinence exceeding 12 months may be critical in terms of recovery, as 65–75 % of AbDs relapse within 12 months of treatment discharge (Sinha 2011). Poor treatment retention and early relapse are associated with higher impulsivity in dependence (Moeller et al. 2001; Patkar et al. 2004; Evren et al. 2012) suggesting that impulsivity mechanisms may be a determinant of sustained abstinence, such that impulsivity measures may be different in those who are able to maintain abstinence longer. There is therefore a need for studies that systematically examine variability in impulsivity associated with varying length of abstinence (extending beyond 12 months).

The evidence for impulsivity in substance dependence is not consistent, especially when we consider the different types of substance dependence (for a review, see Smith et al. 2014). However, there has been little research comparing groups with different dependencies within a single study and the majority of papers have fewer than 30 participants per group (Smith et al. 2014), only providing sufficient power to detect moderate effect sizes in questionnaire and behavioural measures. Therefore, the aim of the present study was to investigate impulsivity measures across different modalities: self-report, behavioural and neuroimaging, in a large number of abstinent participants with a history of dependence on different substances. Although humans use many different substances legally and illegally, heroin, cocaine and alcohol are rated as the most harmful in the UK (Nutt et al. 2010); these were the focus of the present study. We hypothesised that AbDs would show increased impulsivity across all modalities and that this would be more marked in those with dependence on multiple substances compared to those dependent on alcohol alone. A secondary aim was to explore how impulsivity measures vary with length of abstinence, extending to beyond 12 months.

## Methods

This study was conducted as part of the ICCAM Platform Study (www.iccam.org.uk), details of which are reported by Paterson et al. (2015). The protocol was approved by the West London Research Ethics Committee (REC Ref: 11/H0707/9; PI: Prof D.J. Nutt). Non-imaging testing sessions were conducted at three sites: NIHR/Wellcome Trust Imperial Clinical Research Facility, NIHR/Wellcome Trust Cambridge Clinical Research Facility, and Clinical Trials Unit, Salford Royal NHS Foundation Trust. Imaging sessions were conducted in the adjoining centres at Imanova Limited (formerly the GSK Clinical Imaging Centre), Wolfson Brain Imaging Centre, Manchester Translational Imaging Unit (3T MRI Facility), respectively.

### Participants

Participants, including abstinent substance-dependent individuals (AbDs) and controls, were recruited from local NHS addiction services and via advertising on social media, in job centres and libraries. Following written and informed consent, all participants were assessed using the Structured Clinical Interview for DSM-IV to assess for dependence history and checked by a psychiatrist. Exclusion criteria for all participants included lifetime history of psychotic disorder, neurological illness, neurodevelopmental disorder or traumatic head injury. Participants were between 20–65 years old and able to read and write in English. To confirm abstinence on day of testing, all participants completed an alcohol breath test and urine drug screen. Participants were requested to refrain from cannabis use for at least seven days prior to each session but, given the long half-life of cannabinoid metabolites, positive results for cannabinoids were permitted if the participant was not intoxicated or in withdrawal (determined by the psychiatrist conducting the interview). AbDs were abstinent for at least two weeks prior to testing. Nicotine use was not an exclusion criterion in any group as the majority of substance-dependent individuals smoke tobacco.

Of the 179 participants who were consented to the study across the three sites, 14 were excluded immediately at initial screening by the researcher on the basis of obviously failing to meet inclusion criteria (e.g. a history of psychosis). A further 22 participants were subsequently excluded by the clinical committee at a second reviewing stage on the basis of more subtle exclusions (e.g. controls with past cannabis use felt to be approaching dependent levels). This left 143 who were eligible for inclusion in analyses (21.7 % female, aged 25–64, mean 41.66, SD 8.93) comprising 57 control participants with no history of substance dependence (except nicotine), and 86 abstinent substance-dependent participants (see Table 1). The majority of substance-dependent participants had experience with a large number of substances and many met criteria for past dependence on multiple substances. For the purposes of this study, we defined two substance-dependent groups: “alcohol AbD” participants who met DSM-IV criteria for past dependence on alcohol (*n* = 27) and “polydrug AbD” participants who met DSM-IV criteria for past dependence on two or more substances, one of which was alcohol, cocaine or heroin (*n* = 59; see Table 2).

**Table 1:** Demographic data for control, alcohol dependent and polydrug dependent participants

	Control	Alcohol	Polydrug	F or χ^2^	df	*p*
Age (mean, SD)	42.39 (8.74)	45.81 (8.49)	39.05 (8.57)	6.034	2, 140	0.003
IQ (mean, SD)	107.88 (8.83)	104.85 (7.59)	99.32 (10.86)	11.904	2, 140	<0.001
% Female	26.3	22.2	16.9	1.504	2	0.471
% Smokers	56.1	74.1	76.3	5.979	2	0.050

**Table 2:** Substance dependence data for control, alcohol dependent and polydrug dependent participant groups. Data exclude nicotine dependence

		Control	Alcohol	Polydrug	Total
Alcohol dependence	No	57	0	23	80
	Yes	0	27	36	63
Cocaine dependence	No	57	27	14	98
	Yes	0	0	45	45
Opioid dependence	No	57	27	20	104
	Yes	0	0	39	39
Length of abstinence (months)	Alcohol		Polydrug		Polydrug (outlier removed)
Mean (SD)	15.46 (17.76)		20.86 (43.98)		15.63 (18.10)
Median	8.0		9.0		8.5
Range	1.0–79.0		0.5–324.0		0.5–102.0

Three control participants did not complete the behavioural tasks, leaving 54 control participants in the behavioural analysis. Fourteen participants were removed from the GNG imaging analysis due to excessive movement (defined as >20 % volumes with >1 mm movement) or low baseline performance on the GNG imaging task (<85 % Go accuracy), leaving 52 control, 26 alcohol and 51 polydrug participants in the imaging analysis.

### Assessment procedure

#### Clinical variables

Participants were interviewed to ascertain eligibility and group allocation. We also obtained data on their substances of dependence (excluding nicotine) and length of abstinence (see Table 2). For alcohol AbD participants, length of abstinence was calculated from their last use of alcohol to dependent levels. For polydrug AbD participants the multiple substances meant that length of abstinence could only be calculated from the most recent use of any substance of dependence.

#### Self-report questionnaires

Participants completed a battery of impulsivity questionnaires presented in computer format. These included the Barratt Impulsiveness Scale (BIS-11; Patton et al. 1995), Behaviour Inhibition/Activation System (BIS/BAS; Carver and White 1994), the UPPS Impulsive Behaviour Scale (UPPS-P; Whiteside and Lynam 2003) and the Obsessive-Compulsive Inventory Revised (OCI-R; Foa et al. 2002).

#### Behavioural tasks

The Kirby test of delay discounting (Kirby and Maraković 1996) measures the discounting rate; the extent to which the present value of a future reward decreases as the delay to its receipt increases. Hypothetical immediate rewards of £11–80 and delayed rewards of £25–85, with delays of 7–186 days were used. A hyperbolic discount parameter (*k*) score for each participant was generated from the proportion of immediate choices that were made over delayed choices using the method reported by Kirby et al. (1999); and Kirby (2000). Greater discounting, indexed by increasing *k* values, indicates higher levels of impulsivity.

Participants also completed the Stop Signal Task (SST) and the Intra-Extra Dimensional Set Shift (IED) task from the well-validated CANTAB neuropsychological test battery (www.cambridgecognition.com/academic/cantabsuite/executive-function-tests). The SST is a test of motor inhibition, specifically action cancellation (Dalley et al. 2011), at the presentation of an auditory stimulus. A full description is presented by Ersche and Sahakian (2007). The primary outcome is the “stop-signal reaction time” (SSRT), which is the time an individual requires to withhold a response.

The IED is derived from the Wisconsin Card Sorting Task and assesses rule acquisition and reversal, visual discrimination, attentional set formation, maintenance, shifting and flexibility of attention. Primary outcome measures are “total errors” (adjusted for any early terminations), “number of stages completed” and “number of errors at each stage”. A full description is presented in Downes et al. (1989).

#### Functional MR imaging tasks

**Fig. 1:**
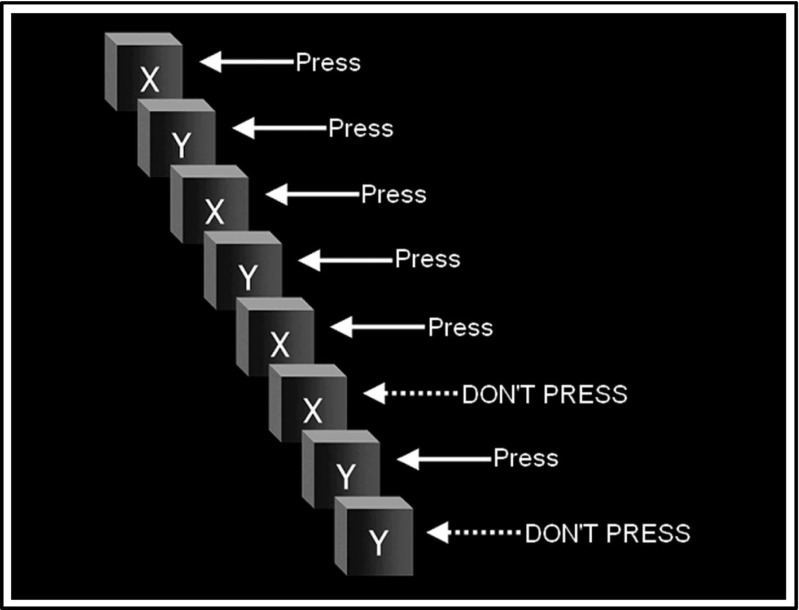
An instruction screen from the Go/NoGo task. Participants are asked to respond as quickly as possible to each letter “X” and “Y” that appears on the screen, except when the letter is the same as the one shown previously

To investigate neural substrates of inhibitory control, participants performed a Go/NoGo (GNG) task whilst being scanned using fMRI (Fig. 1). Participants were presented with a series of individual “X’s” and “Y’s” and asked to respond as quickly as possible to each letter by pressing a button (Go), except when it immediately repeated itself (NoGo). This was an event-related task carried out in two runs of 250 trials, each containing 220 Go trials and 30 NoGo trials. This ratio of frequent Go to rare NoGo trials was used as it is considered a stronger test of pure inhibition than other Go:NoGo ratios (Smith et al. 2014). Each letter was presented for 900 ms and followed by 100 ms inter-stimulus interval of a blank screen. Each run began with a 12-s fixation and lasted for 262 s. Immediately before scanning, participants completed 60 practice trials.

### MR image acquisition

Imaging was carried out at the three sites using a Siemens (Imperial and Cambridge) or a Philips (Manchester) 3T MR scanner. One hundred and thirty one volumes were acquired, comprising 33–36 axial slices of 3 mm thickness, with a TR of 2000 ms, TE of 32 ms and a voxel size of 3 × 3 × 3 mm. In order to maximise cerebral coverage whilst minimising slice thickness and susceptibility artefact, fMRI/EPI acquisition was at +30 degrees to the ACPC line. A T1-weighted structural image was also acquired for use in spatial pre-processing and for examination of any structural abnormalities.

### Data analysis

#### Self-report and behavioural data analysis

Data were analysed using Statistical Package for Social Sciences (SPSS, version 22, www.spss.com) firstly using multivariate analysis of variance (MANOVA) to asses overall group differences. Where significant main effects were found, these were explored with individual univariate analyses of variance, and finally Tukey’s LSD post hoc test when main effects from the univariate analyses were found. Pearson’s Chi-square tests or independent *t* tests were used to assess group differences in demographic variables. In order to explore important relationships between impulsivity measures and length of abstinence, Pearson’s correlations were used within the AbD groups. We also used Pearson’s correlation to explore whether any of the measures where there were significant group differences were related to age, IQ or smoking status in the AbD groups.

#### Image analysis

Imaging data were analysed using statistical parametric mapping (SPM12; Wellcome Trust Centre for Neuroimaging, London, England, http://www.fil.ion.ucl.ac.uk/spm/), implemented in MATLAB (Mathworks 2012, www.mathworks.com). Images were realigned to correct for motion, using the first image as a reference. The structural (T1-weighted) and functional images were then coregistered, followed by spatial normalisation, and were smoothed using a Gaussian kernel filter of 8 × 8 × 8 mm. First level analysis was performed on the contrasts of “Stops” (successful inhibitions) compared to a background of Go responding. Errors of commission were modelled as a contrast of no interest as there were too few for sufficient power. Also modelled as contrasts of no interest were “Sleep”, where there were more than 10 consecutive errors of omission on Go trials, and “False Inhibitions” where successful inhibitions on NoGo trials were immediately preceded by an omitted Go trial.

The second level analysis used a region of interest (ROI) approach based on areas previously identified in the inhibitory control literature as being altered in substance dependence. The areas identified were the right and left inferior frontal gyri (IFG) and the anterior cingulate cortex (ACC), defined by Neuromorphometrics, Inc. (www.neuromorphometrics.com), under academic subscription (Supplementary Figure 1). We extracted the average value of the Stops contrast per person within each ROI and performed group comparisons using independent-samples *t* tests in SPSS. Correlation analyses were conducted by performing one-way ANOVAs with the variables of “NoGo accuracy” and “length of abstinence” entered as separate covariates (*p* < 0.05, Bonferroni corrected for three comparisons). To investigate the relationship with length of abstinence, one polydrug participant was removed due to an outlying length of abstinence score (Table 2) and correlations between BOLD signal in ROIs and length of abstinence were then examined in the alcohol and polydrug AbD groups.

## Results

### Demographics and clinical variables

Participant group demographics can be seen in Table 1, whilst additional dependencies and length of abstinence can be seen in Table 2. One polydrug AbD participant was removed from the length of abstinence analysis due to an outlying length of abstinence of more than two standard deviations from the mean. There were no differences in length of abstinence between alcohol and polydrug AbD participants (t(83) = −0.040, *p* = 0.968).

### Normalisation of data

Initial data screening using Q-Q plots highlighted non-normally distributed scores for IED total errors, Kirby and OCI-R. Therefore, these data were transformed using a log transformation, with the resulting Q-Q regressor plots showing normal distribution.

### Self-report measures

#### Multivariate analysis: group differences on total scores

The effect of group (control, alcohol AbD, polydrug AbD) was analysed using a multivariate analysis of variance (MANOVA) conducted on total scores for each of the self-report measures of BIS-11, UPPS-P, BIS/BAS and OCI-R. Using Pillai’s trace, a significant main effect of group was found (*V* = 0.395, F(10,274) = 6.741, *p* < 0.001).

**Fig. 2:**
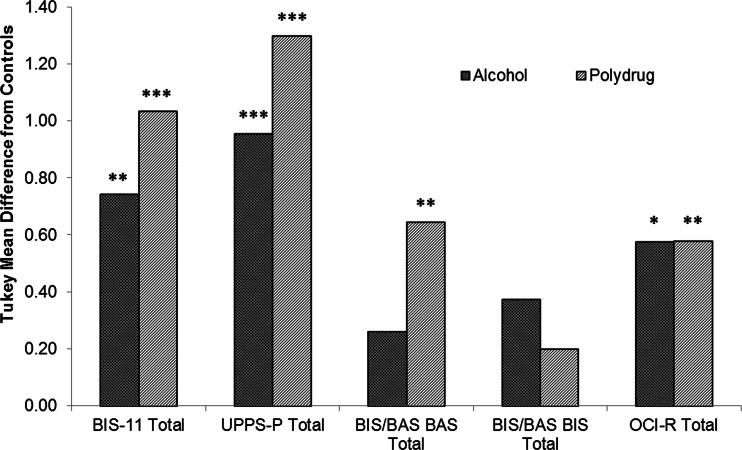
Total scores on self-report measures for alcohol and polydrug AbD groups plotted as their difference from control scores. *p < 0.05, **p < 0.01, ***p < 0.001

This significant main effect allowed separate univariate analyses of variance (ANOVA) to be performed post hoc on each of the outcome variables. These revealed significant group differences on the total scores of BIS-11 (F(2,140) = 22.676, *p* < 0.001), UPPS-P (F(2,140) = 39.284, *p* < 0.001), BAS (F(2,140) = 6.319, *p* < 0.01) and OCI-R (F(2,140) = 6.123, *p* < 0.01). There was no main effect of group on BIS total (F(2,140) = 1.414, *p* = 0.247). Further post hoc analysis using Tukey’s LSD revealed that both alcohol and polydrug AbD groups scored significantly higher on the BIS-11 (alcohol *p* < 0.01; polydrug *p* < 0.001), UPPS-P (alcohol *p* < 0.001; polydrug *p* < 0.001) and OCI-R (alcohol *p* < 0.05; polydrug *p* < 0.01) total scores than controls, whilst only the polydrug AbD group scored significantly higher than controls on BAS (*p* < 0.01) total scores (Fig. 2 and Table 3).

**Table 3:** Mean total scores (and SD) for control, alcohol AbD and polydrug AbD participants on each of the self-report measures

	Control		Alcohol		Polydrug	
BIS-11 total	56.88	(9.45)	66.59	(12.63)	70.41	(11.58)
UPPS_P total	112.70	(19.27)	138.15	(24.72)	147.32	(21.83)
BIS/BAS BAS total	37.35	(5.68)	38.78	(6.01)	40.88	(4.71)
BIS/BAS BIS total	19.09	(4.28)	20.67	(3.96)	19.93	(4.20)
OCI total	7.40	(7.93)	13.59	(13.43)	13.02	(10.05)

#### Group differences on sub-scores

A second MANOVA was conducted to assess for group differences on each of the questionnaire sub-scores. These included the BIS-11 (Attentional Impulsivity, Motor Impulsivity, Non-Planning Impulsivity), UPPS-P (Negative Urgency, Premeditation, Perseverance, Sensation Seeking, Positive Urgency), and BIS/BAS (Drive, Fun, Reward). Using Pillai’s trace, a significant main effect of group was found (*V* = 0.567, F(22,262) = 4.712, *p* < 0.001), allowing for separate univariate ANOVAs to be performed.

These post hoc ANOVAs revealed a significant group difference on BIS-11 Attentional Impulsivity (F(2,140) = 19.128, *p* < 0.001), Motor Impulsivity (F(2,140) = 8.751, *p* < 0.001), and Non-Planning Impulsivity (F(2,140) = 22.801, *p* < 0.001); UPPS-P Negative Urgency (F(2,140) = 35.125, *p* < 0.001), Premeditation (F(2,140) = 7.521, *p* < 0.01), Perseverance (F(2,140) = 16.818, *p* < 0.001), Sensation Seeking (F(2,140) = 6.882, *p* < 0.01), and Positive Urgency (F(2,140) = 26.695, *p* < 0.001); BIS/BAS Drive (F(2,140) = 5.249, *p* < 0.01) and BIS/BAS Fun (F(2,140) = 6.071, *p* < 0.01). There was no main effect of group on BIS/BAS Reward (F(2,140) = 1.766, *p* = 0.175).

Further post hoc analysis using Tukey’s LSD revealed that both alcohol and polydrug AbD groups scored significantly higher than controls on BIS-11 Attentional Impulsivity (alcohol *p* < 0.001; polydrug *p* < 0.001), Motor Impulsivity (alcohol *p* < 0.05; polydrug *p* < 0.001), and Non-Planning Impulsivity (alcohol *p* < 0.05; polydrug *p* < 0.001), whilst polydrug participants also scored significantly higher than alcohol participants on Non-Planning Impulsivity (alcohol *p* < 0.05). Both alcohol and polydrug AbD groups scored significantly higher than controls on UPPS-P Negative Urgency (alcohol *p* < 0.001; polydrug *p* < 0.001), Perseverance (alcohol *p* < 0.01; polydrug *p* < 0.001), and Positive Urgency (alcohol *p* < 0.001; polydrug *p* < 0.001). Polydrug AbD participants scored significantly higher than controls on UPPS-P Premeditation (*p* < 0.001), BIS/BAS Drive (*p* < 0.01) and BIS/BAS Fun (*p* < 0.01), as well as higher than both controls and alcohol AbD participants on UPPS-P Sensation Seeking (control *p* < 0.01; alcohol *p* < 0.05).

### Behavioural tasks

The effect of group (control, alcohol AbD, polydrug AbD) was analysed using a MANOVA on the score outcome measures for each of the behavioural tasks; SSRT, IED total errors and Kirby *k* (Table 4). Using Pillai’s trace, a significant main effect of group was found (*V* = 0.123, F(3,272) = 2.978, *p* < 0.01), allowing for separate univariate ANOVAs to be performed. These post hoc ANOVAs revealed a significant group difference on Kirby *k* (F(2,137) = 6.244, *p* < 0.01), but no significant group differences on SSRT (F(2,137) = 0.822, *p* = 0.442) or IED total errors (F(2,137) = 2.402, *p* = 0.094). Further post hoc analysis using Tukey’s LSD revealed that polydrug participants had significantly higher discounting scores than controls (*p* < 0.01), whilst those of alcohol participants were only marginally higher than controls (*p* = 0.054).

**Table 4:** Mean (SD) scores for each of control, alcohol and polydrug groups on the three behavioural measures of impulsivity

	Control		Alcohol dependent		Polydrug dependent	
SSRT	183.61	(46.73)	190.98	(48.51)	195.31	(50.47)
IED errors (adj.)	26.11	(28.56)	34.52	(36.93)	34.19	(29.77)
Kirby *k*	0.02	(0.02)	0.04	(0.05)	0.04	(0.06)

Additional exploratory analysis was performed on further outcome measures of the SST and IED in order to ensure these were not confounding the results. All participants did not differ on Stop Signal Delay (F(2,137) = 0.369, *p* = 0.692), Successful Stops (F(2,137) = 1.292, *p* = 0.278), or mean Go Reaction Times (F(2,137) = 0.203, *p* = 0.816). There were no significant group differences in the number of IED stages completed (*χ*^2^ = 10.321, *p* = 0.413) nor number of errors at each stage.

### Length of abstinence

In each of the AbD groups, we conducted correlation analyses on the measures where significant differences from controls were observed to explore how these measures related to length of abstinence (Table 5). Shorter length of abstinence was associated with higher scores on BIS-11 Non-Planning Impulsivity (*r* = −0.44, *p* < 0.05), UPPS Negative Urgency (*r* = −0.41, *p* < 0.05) and Premeditation (*r* = −0.38, *p* < 0.05), as well as on BIS/BAS Fun (*r* = −0.38, *p* < 0.05) within the alcohol AbD group. Higher Kirby scores only were found to be associated with shorter length of abstinence in the polydrug AbD group (*r* = −0.40, *p* < 0.01).

**Table 5:** Correlation matrix for length of abstinence with measures of impulsivity

		Alcohol dependent	Polydrug dependent
BIS-11	Total	−0.344	−0.066
	Attention	−0.232	−0.007
	Motor	−0.226	−0.09
	Non-planning	−0.436*	0.054
UPPS-P	Total	−0.455*	−0.198
	Negative urgency	−0.413*	−0.188
	Premeditation	−0.381*	−0.161
	Perseverance	−0.349	−0.086
	Sensation seeking	−0.044	−0.124
	Positive urgency	−0.368	−0.123
BIS/BAS	Drive	−0.257	0.006
	Fun	−0.382*	−0.137
	BAS total	−0.344	−0.078
OCI-R	Total	0.285	−0.010
Kirby	*k*	−0.019	−0.398**

*p < 0.05, **p < 0.01 (2-tailed)

### Correlation analyses associations with age, IQ and smoking status

Correlation analyses were also conducted to assess whether the variables of age, IQ and smoking status might explain the variance in impulsivity measures across groups. We conducted analyses separately within the alcohol and polydrug AbD groups on impulsivity measures where the groups differed significantly from controls. There were no significant correlations (see Supplementary Table 1).

### FMRI analysis

An ANOVA showed no significant group difference in Go RT (F(2,126) = 1.045, *p* = 0.355) or NoGo accuracy (F(2,126) = 0.985, *p* = 0.376). There was, however, a significant difference in Go accuracy (F(2,126) = 4.402, *p* < 0.05). Key values are summarised in Table 6.

**Table 6:** Mean (SD) performance scores for each group on GNG fMRI task

	Control		Alcohol dependent		Polydrug dependent	
Go accuracy (%)	98.58	(2.15)	97.68	(2.71)	96.79	(3.91)
NoGo accuracy (%)	69.49	(15.58)	65.24	(17.65)	65.05	(18.92)
Go RT (ms)	340.18	(72.10)	318.16	(64.75)	341.64	(74.89)

Independent *t* tests between alcohol dependent vs control, polydrug dependent vs control, and alcohol dependent vs polydrug dependent, did not identify any significant BOLD signal changes at the voxel-level Family Wise Error (FWE) corrected threshold of *p* < 0.05. We also used the predefined ROIs of right IFG, left IFG and ACC, none of which identified any significant group differences. Correlation analyses found no significant associations between activation and either NoGo performance or length of abstinence.

### Effects of different dependency histories

Whilst numbers were too small for definitive analysis, we performed an exploratory MANOVA of the 59 polydrug AbD participants to determine whether differences could be attributed to a specific history of dependence on the most commonly used substances in our sample (alcohol, opioids or stimulants). Using Pillai’s trace, a significant main effect of past stimulant dependence was found on total self-report scores (*V* = 0.189, F(5,53) = 2.463, *p* < 0.05), which was found to be due to those with a history of stimulant dependence scoring significantly higher than those with no history of stimulant dependence on the total scores of BIS-11 (F(1,57) = 6.171, *p* < 0.05), UPPS-P (F(1,57) = 7.596, *p* < 0.01), BIS (F(1,57) = 5.886, *p* < 0.05) and OCI-R (F(1,57) = 8.162, *p* < 0.01), but not on BAS (F(1,57) = 0.109, *p* = 0.742). There were no differential effects in those with and without opioid use or alcohol use, and no effects of any of the substances on behavioural or imaging measures.

## Discussion

This investigation compared self-report, behavioural and neural measures of impulsivity in a large abstinent substance-dependent (AbD) population. We found that AbD participants scored higher than controls on most self-report measures, but that alcohol and polydrug dependent groups did not differ from each other. In contrast, there were no group differences on the fMRI GNG task or any of the behavioural measures of impulsivity, except for the Kirby task. These findings add to the growing literature on impulsivity in substance dependence and point to the need for more appropriate and relevant behavioural and neural impulsivity measures for AbD individuals.

### Self-report impulsivity

Both alcohol and polydrug AbD groups were found to be more impulsive than controls across all self-report measures, except the Behaviour Inhibition Scale, in line with previous literature (Ersche et al. 2010, 2011; von Diemen et al. 2008). Although the alcohol and polydrug AbD group scores were not significantly different, there was a trend for the polydrug group to score higher than the alcohol group, perhaps reflecting multiple dependencies (McCown 1988; Semple et al. 2005).

The same pattern was also observed in the subscales of the self-report measures, with both AbD groups scoring significantly higher than control participants whilst not differing from each other. There was one notable exception, with polydrug AbD participants reporting more sensation seeking than both controls and alcohol participants (who did not differ from each other). This may be a result of prevalent stimulant dependence history in the polydrug compared to the alcohol AbD group. Stimulants such as cocaine and amphetamines produce alterations in the mesolimbic dopaminergic system (Volkow et al. 2011), which are associated with trait impulsivity (Dalley et al. 2011). Sensation seeking is particularly associated with stimulant use (Ersche et al. 2010; Mahoney et al. 2015), and the higher scores in polydrug AbD participants in our study could be consistent with their higher levels of stimulant use. This is supported by our exploratory post hoc analysis that divided polydrug AbD participants by their specific past dependencies, in which we found that only when stimulant dependence history, and not opioid or alcohol dependence history, was used as a grouping variable was there a significant difference on self-reported impulsivity. Whilst this suggests that stimulant dependence may be a major determinant of the increased impulsivity in our polydrug AbD group, these findings should be taken with extreme caution due to the unbalanced groups and low power of this analysis.

Although sensation seeking has been highly related to substance dependence (Zuckerman 2007), there is relatively little evidence linking it directly to alcohol dependence (Noël et al. 2011). This is particularly notable in comparison to the wealth of evidence of elevated sensation seeking in stimulant dependence (e.g. Marusich et al. 2011; Mahoney et al. 2015; Ersche et al. 2010; Stoops et al. 2007; Kelly et al. 2006; Patkar et al. 2004). However, the literature seems to focus more on sensation seeking as a risk factor for heavy drinking in adolescence (e.g. Comeau et al. 2001; Shin et al. 2012; Gillespie et al. 2012). Additionally, impulsivity (specifically sensation seeking and lack of premeditation) were found to be more related to illicit substance use in young adults than was hazardous drinking (Shin et al. 2013). To the best of our knowledge there has not been an investigation comparing sensation seeking in stimulant and alcohol dependence (either current or past) within one study.

### Behavioural and neural measures of impulsivity

There were no group differences on neural measures of impulsivity, nor any of the behavioural measures except for the Kirby delay discounting task. One explanation would be that most of our behavioural and neural measures assess state impulsivity, which undergoes change during and immediately after dependence, whilst self-report measures (together with the Kirby task) assess trait impulsivity that is relatively impervious to such changes. Whilst this conclusion is intuitively appealing, it is at odds with evidence of increased SST impulsivity in siblings of stimulant dependent individuals (Ersche et al. 2011) implicating behavioural impulsivity as an endophenotypic trait. It is also important to note that in the present study, length of abstinence was related to BIS-11 Non-Planning Impulsivity, UPPS-P Negative Urgency and Premeditation, as well as BIS/BAS Fun within the alcohol AbD group. Kirby discounting scores were also seen to decrease with length of abstinence within the polydrug AbD group. Thus self-reported (and Kirby discounting) impulsivity appear to decrease with extended abstinence, a pattern not entirely consistent with the hypothesis that self-reported impulsivity is a stable trait. However, the pattern we observed could also be explained by lower levels of trait impulsivity in those able to maintain longer term abstinence. A longitudinal approach would be required to distinguish these possibilities. Another consideration is that self-report measures of impulsivity are more susceptible to bias (Verdejo-García et al. 2008), intentional or otherwise, whilst behavioural tasks are much less prone to this bias. Therefore, this begs the question of whether this bias is driving the self-report impulsivity differences rather than “true impulsivity”. This may be particularly pertinent for abstinent individuals who have learned to inhibit potentially instinctive impulsive behaviours to maintain abstinence.

Of the explicit behavioural measures used here, only the Kirby discounting scores were seen to decrease with extended abstinence within the polydrug dependent group. Another interpretation is that the SST, IED and GNG simply do not capture the most relevant aspects of impulsivity in AbDs, whilst the self-report measures used in this study (as well as the Kirby) are more sensitive to detecting differences. SST, GNG and, to a lesser extent, IED measure inhibition of motor responses and none of these tasks include emotional or motivational components. By contrast, many of the self-report scales for which we found significant group differences measure how an individual reacts to emotional states, such as the UPPS-P Negative Urgency subscale, which is sensitive to differences between dependent individuals (Torres et al. 2013) as well as pathological gamblers (Clark et al. 2012). Similarly, the Kirby behavioural task involves the motivationally salient/ emotionally relevant cue of money. Although “cold cognitive” tests such as the GNG, SST and IED used here are used widely in the addiction literature, and have been found to be sensitive to current and very recent dependence, it may be that more “hot cognitive” tests, that include emotional and motivational dimensions, are required to detect differences in groups able to maintain abstinence.

Therefore, there is a need for validated behavioural measures that assess “affective impulsivity”, in a way that incorporates motivational and emotional components known to be important in both developing substance dependence (Andersen and Teicher 2009; Woicik et al. 2009), and later relapse (Koob and Le Moal 2001). Thus impulsivity tests like the Kirby that involve decision-making about motivationally salient cues may also prove more sensitive than simple motor impulsivity tasks. The multidimensional nature of impulsivity (Evenden 1999; Bari and Robbins 2013) suggests that multiple tasks are required to assess the construct comprehensively and our findings highlight the need for more extensive cognitive and behavioural impulsivity assessment in AbDs.

### Length of abstinence

It is also important to note that in the present study, length of abstinence was related to BIS-11 Non-Planning Impulsivity, UPPS-P Negative Urgency and Premeditation, as well as BIS/BAS Fun within the alcohol AbD group. Kirby discounting scores were also negatively correlated with length of abstinence within the polydrug AbD group. Thus self-reported (and Kirby discounting) impulsivity appears to decrease with extended abstinence, a pattern not entirely consistent with the hypothesis that self-reported impulsivity is a stable trait. However, the pattern we observed could also be explained by lower levels of trait impulsivity in those able to maintain longer term abstinence. Unfortunately, the nature of the design of this study is such that we cannot determine whether impulsivity predated or is a consequence the individuals’ drug use. A longitudinal approach would be required to distinguish these possibilities.

Nevertheless, length of abstinence is an important point of variation between previous studies in this area. Ersche et al.’s (2011) stimulant dependent individuals who showed increased impulsivity were not abstinent. Recently Naim-Feil et al. (2014) reported persisting impulsivity in abstinent alcohol dependent participants with a mean duration of abstinence of approximately 6 months. In our sample, mean length of abstinence was over 12 months and may have allowed individuals time for significant cognitive improvement. Although there is some evidence of improvement of inhibitory control with abstinence (Hopwood et al. 2011; Morie et al. 2014), including functioning of relevant neural circuits (Bell et al. 2014), there is also evidence of an improvement in executive functioning (Schulte et al. 2014; Stavro et al. 2013; Sullivan et al. 2000), into which inhibitory control can be included as a wider construct (see also a review by Fernández-Serrano et al. 2011). Consistent with the idea of recovery of executive functions, including inhibitory control, we found less impulsive performance on the Kirby task to be associated with longer abstinence in the polydrug AbD group.

Alternatively, including only stably abstinent participants may have biased our sample towards individuals with lower cognitive impulsivity, since the most impulsive individuals prone to early relapse will have been excluded. Early relapse and poor treatment retention are associated with higher impulsivity in stimulant dependence, (Moeller et al. 2001; Patkar et al. 2004) and alcohol dependence (Evren et al. 2012), although not in opioid dependence (Passetti et al. 2008). Nevertheless, the correlations between self-report measures and length of abstinence were relatively weak and do not explain all of the variance. Self-reported impulsivity is thus elevated even in those with long-term abstinence.

### Limitations

A limitation of this study is that the polydrug AbD participants had past dependence on a wide range of substances with substantially different individual profiles of dependent and non-dependent use. Whilst the cohort was representative of the substance-dependent population in the UK, this heterogeneity precluded systematic analysis of the contribution of different substances to the effects observed. For example, the specific contribution of a history of stimulant dependence to impulsivity was impossible to isolate. Poly-substance use is an important issue in addiction research, particularly the potential distinction of dependence on stimulants from other substances, with some studies suggesting that opioid dependence is behaviourally distinct from stimulant dependence (Badiani et al. 2011; Vassileva et al. 2014). Thus, for example, high impulsivity in opioid use has been suggested to be a result of drug use rather than a risk factor in the development of dependence (Harty et al. 2011; Schippers et al. 2012), by contrast to stimulant dependence where high impulsivity is a well-established risk factor (Dalley et al. 2011; Ersche et al. 2010). In addition, a recent paper by Whelan et al. (2014) showed that impulsivity played a relatively minor role in the development of alcohol dependence. However, the clinical reality of drug addiction in the UK is a very high prevalence of poly-substance use and dependence; a meta-analysis by Smith et al. (2014) noted that there was little consistency across studies in the recording of the amount or length of drug use, pointing out that many findings need to be considered with caution. Thus, it is difficult to study single dependencies empirically and the practical clinical relevance of doing so is questionable, as polydrug dependency is the more common clinical challenge.

As a result of these difficulties in recruiting individuals with single dependencies (excluding nicotine), our alcohol group was relatively small (*n* = 26), only providing sufficient power to detect moderate effect sizes (Smith et al. 2014). It may be more valuable for future investigations to consider all AbD participants as one group and investigate their different profiles that are not based on the substances used.

Another limitation of this study is that the groups differed on the measures of age, IQ and smoking status. Nevertheless, these variables were not found to correlate with any of the group differences identified in the present results, implying that they were not responsible for the differences found. It is perhaps surprising that there were such weak correlations between performance on the cognitive tasks and IQ. This is in line with some previous studies, but not all; for example a large recent study reported a significant correlation between IQ and delay discounting (de Wit et al. 2007). However it should be noted that other aspects of impulsivity were not related to IQ in that study, and furthermore the effect was observed in a much larger sample than that studied here and with a mean IQ markedly higher.

In addition, the high upper age range of participants within this study (65 years) may have introduced an age-related bias in impulsivity. There is substantial evidence of brain atrophy with older age, for example the rate of cortical atrophy increases to 0.35 % a year after the age of 52, compared to 0.12 % in young adulthood, ventricle size expands at rate of 4.25 % after 70 years compared to 0.43 % in young adulthood, whilst the frontal lobes, which are involved in inhibitory control, show the steepest decline (for a review, see Dennis and Cabeza 2011). In addition, older adults show more compensation for poorer inhibitory control that declines with increasing age (Nielson et al. 2002), as well as poorer motor control (Levin et al. 2014). However, neither AbD group in the present study differed significantly in age from the control group (although the alcohol and polydrug groups differed) nor none of the variables showing group differences were correlated with age.

## Conclusion

The present study suggests that the self-report measures used are more sensitive to detecting impulsivity in long-term abstinent individuals than the behavioural or neuronal measures. Our findings suggest the importance of developing behavioural measures that assess different aspects of impulsivity rather than simple motor response inhibition, alongside corresponding behavioural challenges to use in conjunction with fMRI. A complementary approach may be to reconsider the grouping of individuals in studies of dependence, with a shift of emphasis to cognitive endophenotypes rather than specific substances used. Such an approach would obviate the problem of categorising individuals with complicated drug use and dependence histories, and would have implications for optimising approaches to treatment and prevention based on cognitive profiles.

## Electronic supplementary material

Below is the link to the electronic supplementary material.

ESM 1 (DOCX 226 kb)

ESM 2 (DOCX 18 kb)
