# Supplementary material for: Impulsivity in abstinent alcohol and polydrug dependence: a multidimensional approach
Source: Psychopharmacology (Berl). 2016 Feb 25;233:1487–99. doi: 10.1007/s00213-016-4245-6 (PMC4819593; doi:10.1007/s00213-016-4245-6)
Supplement: Supplementary file 1 — (DOCX 226 kb) [file 213_2016_4245_MOESM1_ESM.docx]

**Supplementary Materials**


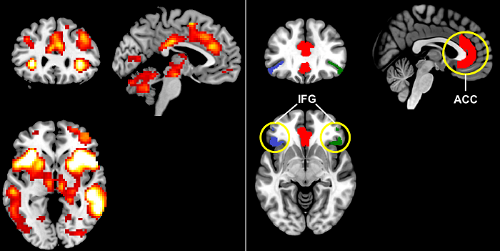


**a**

**b**

*Supplementary Figure 1: a) Significant pattern of task-induced activation for successful inhibitions (p<0.05, Family-wise error) b) Region of interest masks, defined by Neuromorphometrics, Inc. (*[*www.neuromorphometrics.com*](http://www.neuromorphometrics.com)*), under academic subscription, including the anterior cingulate cortex (ACC), rightand left inferior frontal gyri (IFG).*
