# Supplementary material for: Impulsivity in abstinent alcohol and polydrug dependence: a multidimensional approach
Source: Psychopharmacology (Berl). 2016 Feb 25;233:1487–99. doi: 10.1007/s00213-016-4245-6 (PMC4819593; doi:10.1007/s00213-016-4245-6)
Supplement: Supplementary file 2 — (DOCX 18 kb) [file 213_2016_4245_MOESM2_ESM.docx]

**Supplementary Materials**

| ***Supplementary Table 1:*** *Correlation matrix for variables of age, IQ and smoking status with measures of impulsivity presented separately for alcohol AbDs and polydrug AbDs.* | | | | | | | | | | |
| --- | --- | --- | --- | --- | --- | --- | --- | --- | --- | --- |
|  |  |  |  |  |  |  |  |  |  |  |
|  |  |  | **Alcohol** | | |  | **Polydrug** | | |  |
|  |  | | **Age** | **IQ** | **Smoking** |  | **Age** | **IQ** | **Smoking** |  |
|  | **Kirby** | **r** | .154 | -.006 | .004 |  | -.075 | -.177 | .115 |  |
|  |  | **p** | .444 | .977 | .985 |  | .572 | .179 | .384 |  |
|  | **BIS-11 Total** | **r** | -.285 | .041 | -.163 |  | .121 | .049 | .048 |  |
|  |  | **p** | .149 | .837 | .418 |  | .361 | .713 | .721 |  |
|  | **UPPS-P Total** | **r** | -.007 | -.043 | -.007 |  | .184 | .067 | .029 |  |
|  |  | **p** | .972 | .833 | .973 |  | .163 | .613 | .830 |  |
|  | **BIS/BAS BAS Total** | **r** | .106 | .041 | .207 |  | .068 | -.080 | -.245 |  |
|  |  | **p** | .598 | .841 | .301 |  | .611 | .549 | .062 |  |
|  | **OCI-R Total** | **r** | .200 | .269 | .031 |  | -.077 | .121 | .248 |  |
|  |  | **p** | .318 | .175 | .879 |  | .564 | .361 | .058 |  |
|  |  |  |  |  |  |  |  |  |  |  |
